# CRISPR-edited CART with GM-CSF knockout and auto secretion of IL6 and IL1 blockers in patients with hematologic malignancy

**DOI:** 10.1038/s41421-021-00255-4

**Published:** 2021-04-27

**Authors:** Yan Yi, Xiaoshan Chai, Liping Zheng, Yongjing Zhang, Jiankai Shen, Biliang Hu, Guangshi Tao

**Affiliations:** 1 The Second Xiangya Hospital of Central South University, Changsha, Hunan 410011 China; 2 Hunan Key Laboratory of Tumor Models and Individualized Medicine, Changsha, Hunan 410011 China; 3 Celledit, Worcester, MA 01606 USA; 4 Siweikang Therapeutics, Changsha, Hunan 410013 China

**Keywords:** Cancer therapy, Immunology

## Abstract

Revolutionary CART therapy still faces the challenge of severe cytokine release syndrome (CRS). While IL6 and IL1 have been demonstrated as essential contributors, GM-CSF is one of the most abundant inflammatory cytokines secreted by CART and has also been suggested in contributing to CRS. To minimize GM-CSF production from CART to reduce its associated toxicity, we conducted a pilot study (ChiCTR2000032124) of CRISPR-edited GM-CSF knockout (KO) in CART secreting anti-IL6 scFv and IL1RA, with additional TCR KO for tracing edited CART. The initial results of three patients (1 Non-Hodgkin lymphoma (NHL) and 2 multiple myelomas (MMs)) are summarized as: 3/3 complete response, 2/3 none CRS, 1/3 grade 2 CRS, and 0/3 neurotoxicity. The analysis revealed low levels of GM-CSF, IL6 and IL1B at the time of interferon-gamma (IFNG) peaks, and elevated IL1RA. We also observed significant expansion of CD3^–^ CART during treatment and no aberrant expansion of CD3^–^ CART in the follow-up. Re-expansion of CD3^–^ CART was observed in two patients while recurring CD19^+^ cells were eradicated in the patient with NHL. In summary, our study supported the safety and durable potency of CRISPR-edited CART in patients, providing a novel platform for developing autologous or allogeneic CART to minimize GM-CSF-associated toxicity in addition to autonomous IL6/IL1 blockade.

## Introduction

Chimeric Antigen Receptor engineered T cells (CART) have been emerging as one of the revolutionary immunotherapies for patients with hematologic malignancy^1^. However, severe cytokine toxicity and neurotoxicity remain as one of the major challenges in clinical practice of CART therapy, therefore, urging scientists to understand the mechanisms underlying cytokine toxicity and explore novel strategies to resolve it. The central axis of CART therapy is CAR-expressing T cells specifically recognizing antigen on the surface of tumor cells to elicit cytolytic activity of tumor cells. Once infused back to the patient, CART cells first engage with tumor antigen to lyse tumor cells and simultaneously secrete high level of various cytokines. The lysis of tumor cells and CART-secreted cytokines then activate bystander immune cells in the patient, especially macrophage and monocyte to secrete large amount of cytokines, including IL6. The accumulation of cytokines secreted by CART and bystander immune cells converges into cytokine storm while CART cells expand significantly and numerous tumor cells are eliminated. During this process, it is earlier for CART cells to secret cytokines than bystander immune cells, and CART secreted cytokines play an important role in activating bystander immune cells to secrete additional inflammatory cytokines. Therefore, we propose that the cytokines secreted by CART cells can be categorized as the “first wave” of cytokines during cytokine storm, while bystander immune cells produced cytokines as the “second wave” of cytokines.

**Fig. 1:**
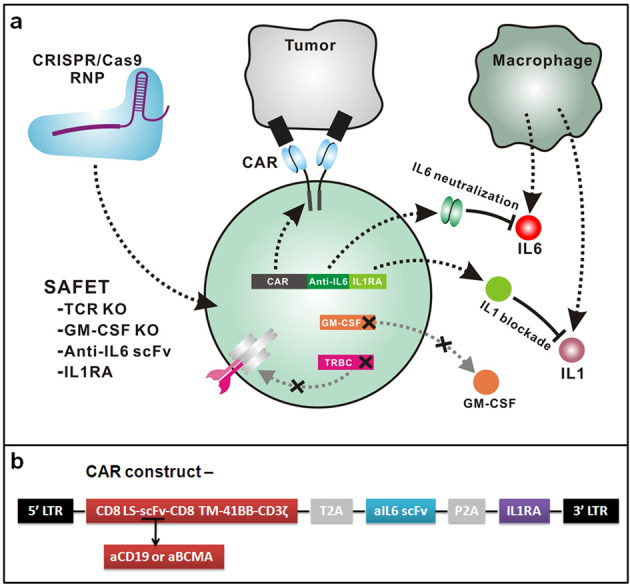
The design of SAFET platform for CART cell therapy. **a** Scheme of CRISPR-edited GM-CSF KO CART cells secreting IL6 and IL1 blockers. Patient-derived T cells were engineered with CRISPR/Cas9 RNP for GM-CSF/TCR KO and transduced with lentivector encoding 2nd generation 41BBζ CAR and anti-IL6 scFv and IL1RA for blocking IL6/IL1 signalings. **b** The construct of lentivector for co-expressing CAR and cytokine blockers.

A feasible strategy to resolve cytokine toxicity should be able to significantly reduce the downstream signaling of key cytokines from both the first and second waves of cytokine storm, which are involved in cytokine toxicity and neurotoxicity but do not play an essential role in CART therapeutic efficacy. In regard to the cytokines from the first wave secreted by CART, genetic editing is very appealing to directly and permanently disrupt the endogenous genes for encoding the cytokines of interest. A recent study by Dr. Carl June’s team demonstrated the safety and efficacy of CRISPR-engineered T cells in treating cancer patients, paving the basis for clinical application of CRISPR gene editing in T cell-mediated cellular immunotherapy^2^. As for the second wave of cytokines secreted by patient immune cells, autonomous co-expression of a cytokine-specific blocker can be incorporated into the gene cassette for encoding CAR. Among the first wave of cytokines, GM-CSF serves a promising target since GM-CSF has been well recognized in activating monocytes and macrophages^3^. Moreover, previous studies by Dr. Saad Kenderian’s team and Dr. Julien Valton’s team demonstrated that GM-CSF inhibition reduced cytokine release syndrome in animal models^4,5^, supporting that GM-CSF KO will be a promising strategy in reducing CRS severity. Among the second wave of cytokines, IL6 and IL1 have been suggested to contribute to severe CRS and neurotoxicity in mouse models^6,7^. Notably, IL6 level significantly increased in the patients with severe CRS, and Tocilizumab has been approved by FDA to treat CRS during CART treatment. Therefore, we designed a novel safe T cells (designated as SAFET) platform for reducing cytokine toxicity, consisting of CRISPR-edited KO of GM-CSF and autonomous co-expression of IL6 and IL1 blockers in CART, referred as CART-aIL6/IL1RA with GM-CSF/TCR KO (as illustrated in Fig. 1a, b).

Here in this study, we present the initial clinical results (ChiCTR2000032124) of one patient with Non-Hodgkin lymphoma (NHL) and two patients with multiple myeloma (MM) treated with CART-aIL6/IL1RA with GM-CSF/TCR KO.

## Results

### Successful KO of GM-CSF through CRISPR /Cas9 gene editing

**Fig. 2:**
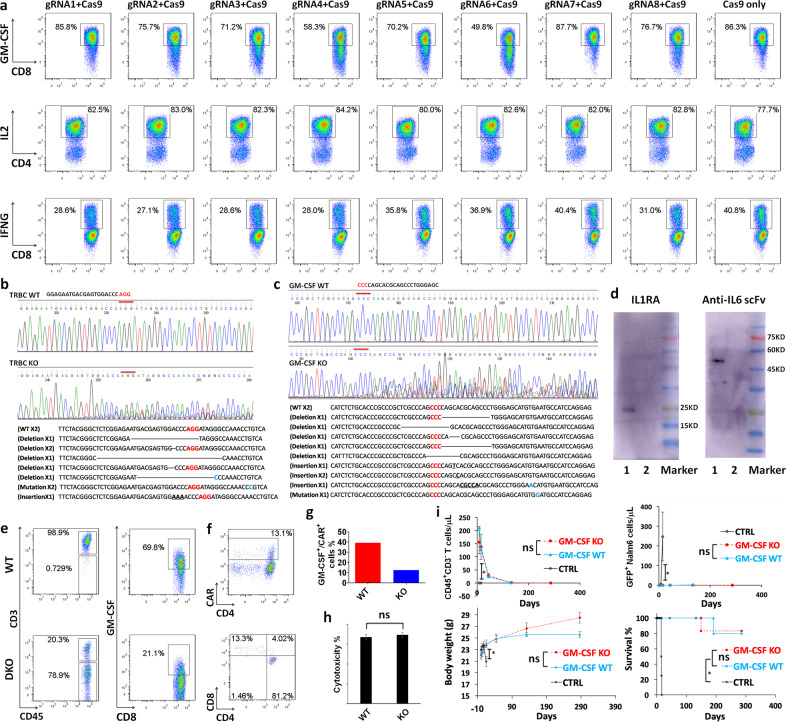
Screening of sgRNA for CRISPR-edited KO of GM-CSF and anti-CD19 CART-aIL6/IL1RA with GM-CSF/TCR KO in xenograft study. **a** sgRNA candidates targeting the first exon of GM-CSF were electroporated with Cas9 protein in normal donor T cells and subsequently tested for cytokine expression via intracellular staining. **b**, **c** Normal donor T cells were engineered with CRISPR/Cas9 RNP for TCR KO (**b**) or GM-CSF KO (**c**). The edited T cells were then collected for PCR amplification of a region spanning the PAM (NGG) site targeted by sgRNA. Amplified PCR products were Sanger sequenced. PCR products amplified from edited T cells were also ligated to a pUC-19 vector and subsequently transformed into competent cells. Single colonies were picked up for preparation of plasmid DNA, which were then Sanger sequenced to reveal the genetic alteration after CRISPR editing. **d** Anti-CD19 CART co-expressing IL1RA and anti-IL6 scFv fused to GST/His tag were cocultured with Nalm6-GFP cells and the culture supernatant was collected for WB analysis with antibodies against human IL1RA and His tag (Lane 1). Non-transduced T cells were included as control (Lane 2). **e**, **f** Normal donor T cells were engineered with CRISPR/Cas9 RNP for GM-CSF/TCR KO and transduced with lentivector encoding 2nd generation 41BBζ CAR and anti-IL6 scFv and IL1RA: **e** the KO efficiency of TCR and GM-CSF; **f** CAR frequency and CD4/CD8 subpopulations in edited CD3^–^ T cells. **g** Anti-CD19 CART cells with GM-CSF KO or WT were stimulated with Nalm6-GFP cells and analyzed by intracellular staining. The representative result of two independent experiments was shown. **h** Cytotoxicity of anti-CD19 CART-aIL6/IL1RA with GM-CSF/TCR KO or only TCR KO. Statistical analysis by Student’s *t* test showed no significance (ns). **i** Antitumor efficacy of anti-CD19 CART-aIL6/IL1RA with GM-CSF/TCR KO in xenograft model. Statistical analysis by Student’s *t* test was conducted for body weight (BW), GFP^+^ Nalm6 cell number, and CD45^+^CD3^–^ T cell number, with Log-Rank test for survival curves. * Indicates statistical significance with *P* < 0.05 and ns indicates no significance.

In this study, we proposed a novel platform for reducing cytokine toxicity, consisting of CRISPR-edited KO of GM-CSF and autonomous co-expression of IL6 and IL1 blockers in CART, referred as CART-aIL6/IL1RA with GM-CSF/TCR KO (as illustrated in Fig. 1a, b). In order to disrupt the gene for encoding GM-CSF, we designed the single guide RNA (sgRNA) sequences for targeting the PAM motifs located in the first exon of GM-CSF gene according to the principle outlined in the handbook of sgRNA synthesis kit by Thermo Fisher Scientific. Edited T cells were analyzed by intracellular staining after activation with PMA and inomycin to assess the efficiency of gene editing against GM-CSF. The results indicated that sgRNA6 was the most efficient in disrupting GM-CSF expression, as compared to that in the T cells with Cas9 protein only (Fig. 2a). Meanwhile, the analysis showed that there were no significant differences in the percentage of IL2- or IFNG-secreting T cells, as edited by sgRNA6 or Cas9 protein only. In sum, these results suggest that CRISPR gene editing could successfully knockout the gene of GM-CSF in a specific manner, without affecting the expression of IFNG and IL2.

In order to confirm the gene editing of GM-CSF and TCR by CRISPR at the molecular level, we conducted Tracking of Indels by Decomposition (TIDE) in normal donor T cells engineered with CRISPR/Cas9 ribonucleoprotein (RNP) for TCR KO (Fig. 2b) or GM-CSF KO (GM-CSF sgRNA6, Fig. 2c). The edited T cells were then collected for PCR amplification of a region spanning the PAM (NGG) site targeted by sgRNA. Amplified PCR products were Sanger sequenced, showing overlapping signal peaks around the targeted PAM motifs. PCR products amplified from edited T cells were also ligated to a pUC-19 vector and subsequently transformed into competent cells. Single colonies were picked up for preparation of plasmid DNA, which were then Sanger sequenced to reveal the genetic alterations after CRISPR editing as shown in Fig. 2b, c. These results indicated that GM-CSF sgRNA6 with Cas9 efficiently edited the specific site in GM-CSF gene locus and therefore was chosen for further investigation.

### CRISPR-edited anti-CD19 CART-aIL6/IL1RA with GM-CSF/TCR KO efficiently eradicated tumor cells in xenograft model

Having demonstrated efficient gene disruption of GM-CSF by CRISPR/Cas9, we then engineered CART for autonomous co-expression of IL1RA and anti-IL6 scFv with CAR as depicted in Fig. 1b. To this aim, western blotting (WB) analysis was conducted to confirm the secretion of IL1RA and anti-IL6 scFv. In order to facilitate detection by WB, a GST/His tag was fused to anti-IL6 scFv. Anti-CD19 CART cells were cocultured with Nalm6-GFP cells and the culture supernatant was collected for WB analysis (Fig. 2d, Lane 1), showing a clear band stained by anti-human IL1RA between 15KD and 25KD, and a clear band stained by anti-His tag between 45KD and 60KD. These results confirmed that IL1RA and anti-scFv could be efficiently synthesized and secreted.

Next, we moved onto the engineering of CART co-expressing IL6/IL1 blockers with GM-CSF/TCR KO, with T cells derived from a normal donor. The results showed successful editing of TCR and GM-CSF and CAR transduction (Fig. 2e, f), and the expanded CART cells were subsequently purified with anti-CD3 beads through MACS column to remove the remaining CD3^+^ T cells. Anti-CD19 CART cells with GM-CSF KO or wildtype (WT) were stimulated with Nalm6-GFP cells and analyzed by intracellular staining, revealing reduced percentage of GM-CSF production (Fig. 2g). More importantly, the CART-aIL6/IL1RA with GM-CSF/TCR KO targeting CD19 displayed comparable cytotoxicity against CD19^+^ Nalm6 leukemia cells stably expressing GFP as the GM-CSF wild-type counterpart (Fig. 2h), proving that gene editing of GM-CSF did not affect the killing capacity of CART in vitro. Next, we continued to investigate whether these modified CART cells would be potent in killing tumor cells in vivo. In the xenograft study of NSG mice bearing Nalm6 leukemia cells expressing GFP, we demonstrated that mice treated with anti-CD19 CART-aIL6/IL1RA with GM-CSF/TCR KO significantly prolonged survival by completely eliminating leukemia cells (Fig. 2i). The numbers of CD45^+^CD3^–^ T cells in the mice treated with CART decreased over time after tumor cells were eradicated, suggesting the CART with CRISPR gene editing was safe in mouse models without transforming into tumorigenic state.

### Ex vivo CRISPR editing of patient-derived T cells

**Fig. 3:**
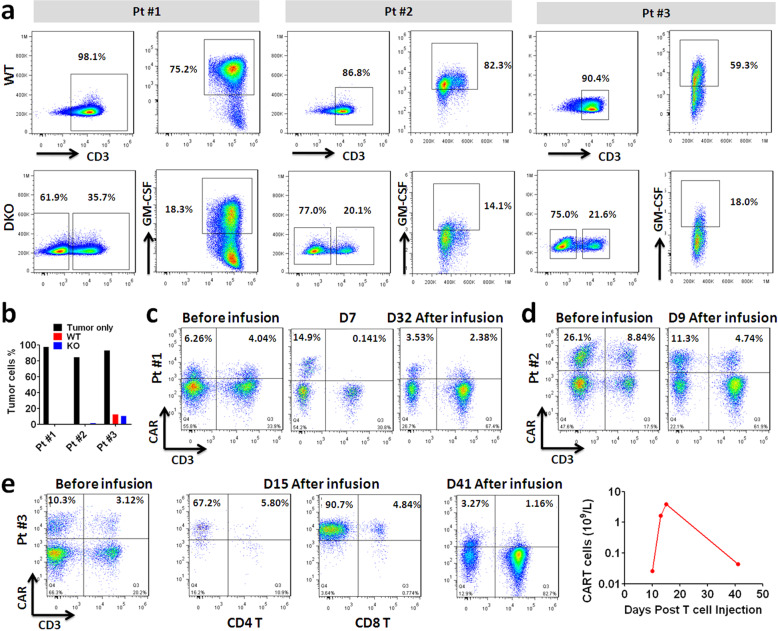
Expansion of CRISPR-edited autologous anti-CD19 or anti-BCMA CART-aIL6/IL1RA with GM-CSF/TCR KO in patients. **a** Efficiency of GM-CSF and TCR KO in CART-aIL6/IL1RA during ex vivo expansion. **b** Cytotoxicity of anti-CD19 or anti-BCMA CART-aIL6/IL1RA with GM-CSF/TCR KO or WT counterpart. **c**–**e** Frequencies of CRISPR-edited CD3^–^ CART cells in patient #1 (**c**), patient #2 (**d**), patient #3 (**e**), before and after infusion.

In the early phase of translation study, one patient with refractory NHL and two patients with refractory MM were enrolled to evaluate the safety and efficacy of CART-aIL6/IL1RA with GM-CSF/TCR KO. During ex vivo production, patient T cells were transduced with 3rd generation lentivector encoding the 2nd generation CAR consisting of 41BB and CD3ζ signalings linked with IL6/IL1 blockers. In addition, T cells were subsequently electroporated with Ribonucleoprotein (RNP) complex of sgRNA targeting GM-CSF and TRBC with recombinant Cas9 protein. Afterward, T cells were analyzed for CAR expression, gene editing efficiency, and tumor lysis through flow cytometry. The results confirmed that gene editing of GM-CSF and TCR was achieved (Fig. 3a), and the edited CART displayed efficient cytotoxicity against Nalm6 leukemia cells or RPMI 8226 cells (Fig. 3b). These results supported the successful ex vivo generation of CART-aIL6/IL1RA with GM-CSF/TCR KO.

### Significant expansion of CRISPR-edited CART in patients

After infusion, blood samples were collected to assess CART proliferation by flow cytometry and qPCR. The results indicated high frequencies of CD3^–^ CART cells in all three patients (Fig. 3c–e). The ratio of CD3^–^ to CD3^+^ CART was comparable before and after infusion in all patients in long-term follow-up. Of particular, a tremendous proliferation of CD3^–^ CART was observed in patient #3 (Pt. #3). Total expansion fold of CRISPR-edited CD3^–^ CART at peak time D15 after the infusion was estimated to be ∼3950 folds. These results demonstrated that GM-CSF/TCR KO did not impair CART proliferation in the patients, which is pivotal to the clinical efficacy of tumor eradication.

### All three patients treated with CRISPR-edited CART achieved complete response

**Fig. 4:**
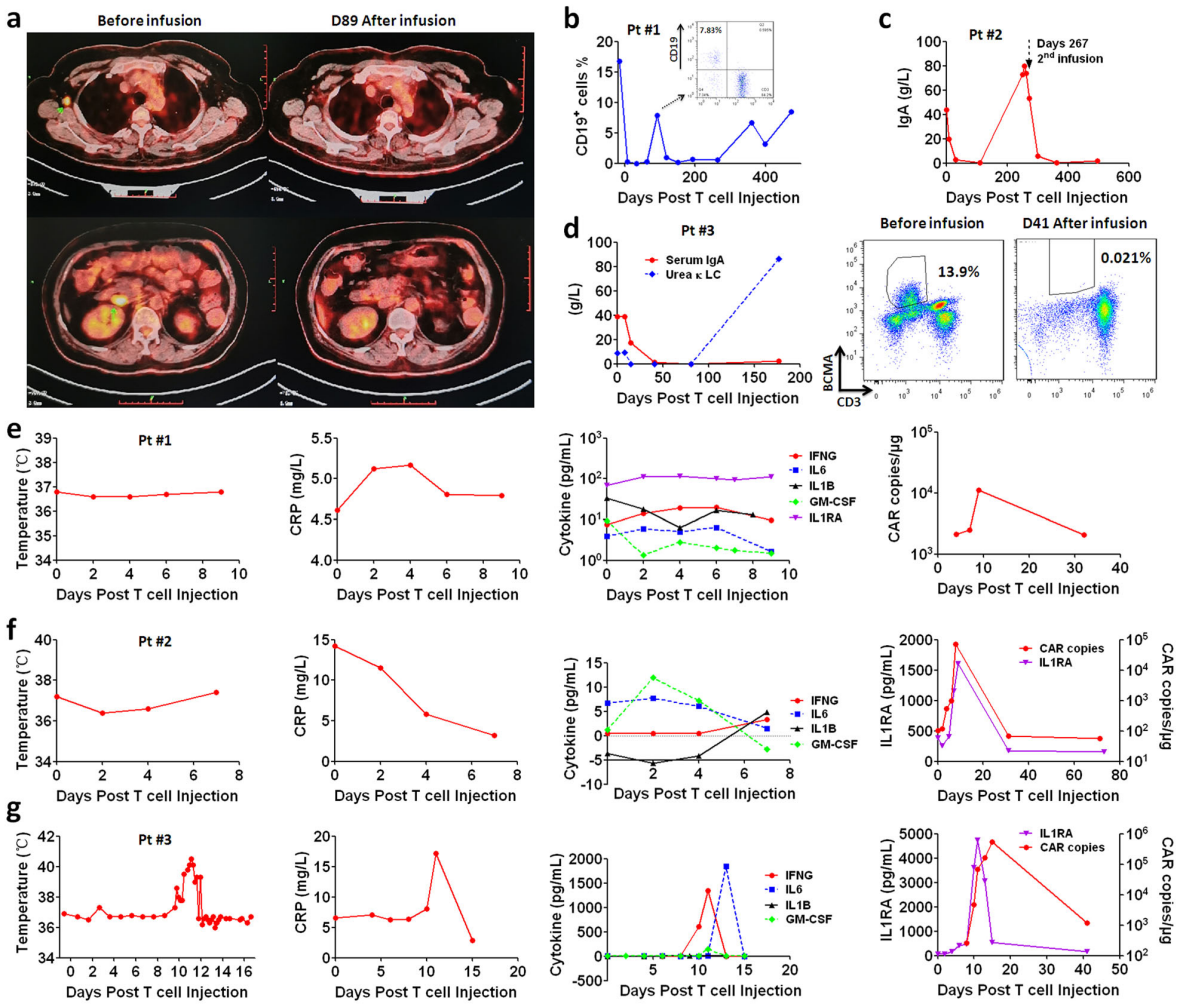
Complete response was achieved in all three patients after treatment with CRISPR-edited anti-CD19 or anti-BCMA CART-aIL6/IL1RA with GM-CSF/TCR KO. **a** PET-CT imaging of primary tumor sites in right armpit and retroperitoneum in patient #1 before and after treatment, as pointed by the green arrow. **b** Frequencies of CD19^+^ B cells in the blood of patient #1 before and after infusion. **c** Changes of serum IgA in patient #2. Patient was given the 2nd infusion of CART co-expressing IL6 and IL1 blockers with GM-CSF/TCR WT when IgA bounced back to aberrant level. **d** Changes of serum IgA and Urea κ Light Chain (LC) in patient #3 (left panel), and frequencies of BCMA^+^ cells in the blood before and after infusion (right panel). **e**–**g** Changes of body temperature, cytokines (CRP C-reactive protein), and CAR vector copes in patient #1 (**e**), patient #2 (**f**), patient #3 (**g**) treated with CRISPR-edited anti-CD19 or anti-BCMA CART-aIL6/IL1RA with GM-CSF/TCR KO.

All three patients reached complete response after treatment, as summarized in Table 1. In patient #1, PET-CT imaging indicated eradication of primary tumor sites (Fig. 4a) and B cell aplasia was maintained for long term (Fig. 4b). In patient #2 and #3, aberrant levels of IgA decreased to normal levels after treatment (Fig. 4c, d). In patient #3, BCMA^+^ tumor cells were detected at 13.9% before treatment, which disappeared after treatment at Day 41 post infusion, further confirming complete response. So far, complete remission is still maintained in patient #1, whereas patient #2 and #3 developed relapses with IgA and Urea κ Light Chain (LC) bouncing back, suggesting antigen-positive and negative relapses, respectively (Fig. 4c, d). In regard to patient #2, there was an extra frozen dose of CART cells co-expressing IL6 and IL1 blockers with GM-CSF/TCR WT, which were then infused back. Notably, complete remission was re-established in patient #2 for long-term survival. These results supported that gene editing of GM-CSF KO did not impair CART therapeutic efficacy. Furthermore, lack of TCR did not comprise CART cytotoxicity, proliferation, or therapeutic efficacy in patients.

**Table 1:** Summary of the patient characteristics.

Patient #	1	2	3
**Gender**	Female	Female	Male
**Age (years)**	49	59	45
**Diagnosis**	Non-Hodgkin’s lymphoma(Transformation of follicular lymphoma to diffuse large B-cell lymphoma)	IgA lambda multiple myeloma	IgA kappa multiple myeloma
**Clinical sites**	Cervical/axillary lymph nodes, abdominal masses	Bone marrow	Bone marrow, lytic bone lesions
**Prior therapy**	R-CHOP(Rituximab, cyclophosphamide, epirubicin, vincristine, prednisone) FC(Fludarabine, cyclophosphamide)	Bortezomib, dexamethasone, thalidomide, cyclophosphamide, etoposide, lenalidomide, Ixazomib (six lines)	Bortezomib, dexamethasone, thalidomide, cyclophosphamide, lenalidomide, vincristine, epirubicin, methylprednisone, cisplatin, etoposide (eight lines)
**Prior surgery**	Splenectomy	None	None
**Dose (X10**^**8**^**)**	0.5	0.5	0.05
**Response**	CR	CR	CR
**CRS grading**	0	0	2
**Fatigue**	No	No	Yes
**Fever**	No	No	Yes
**Hypoxia or dyspnea**	No	No	Yes
**Hypotension**	No	No	No
**Sinus tachycardia**	No	No	Yes
**Altered mental status**	No	No	No
**Infection**	No	No	No
**Hematologic AE**	No	No	No
**Electrolyte AE**	No	No	No
**Renal AE**	No	No	No
**Gastrointestinal AE**	No	No	No

NHL, non-Hodgkin lymphoma; MM, multiple myeloma; CR, complete response; AE, adverse event.

### CRISPR-edited CART-aIL6/IL1RA mediated minimal cytokine toxicity and no neurotoxicity

After infusion, patients were closely monitored for clinical signs of cytokine release syndrome (CRS) and neurotoxicity. No CRS was observed in patient #1 and #2, and grade 2 CRS was observed in patients #3, while none neurotoxicity was observed in any patient. Cytokine levels were analyzed to better understand the CRS during treatment, revealing low level of IFNG in patients #1 and #2, and high level of IFNG in patient #3 (Fig. 4e–g). It is notable that IL6 was maintained at very low level in all three patients during CRS period. Analysis of IL1RA showed close correlation between CART expansion and IL1RA secretion, suggesting that CART cells could efficiently synthesize IL1RA and anti-IL6 scFv while eradicating the tumor cells. The analysis also revealed low level of IL1B in all three patients, supporting that CART secreted IL6/IL1 blockers could significantly reduce IL6 and IL1 associated cytokine toxicity and neurotoxicity. Interestingly in patient #3 after CRS already ended, IL6 level dramatically increased after the peaks of IFNG and IL1RA, and quickly decreased in the following 2 days without causing any further toxicity. These results demonstrated that the CART with GM-CSF/TCR KO and secreting IL6/IL1 blockers could significantly reduce cytokine toxicity and minimize neurotoxicity.

### Persistense of CRISPR-edited CART in patients

**Fig. 5:**
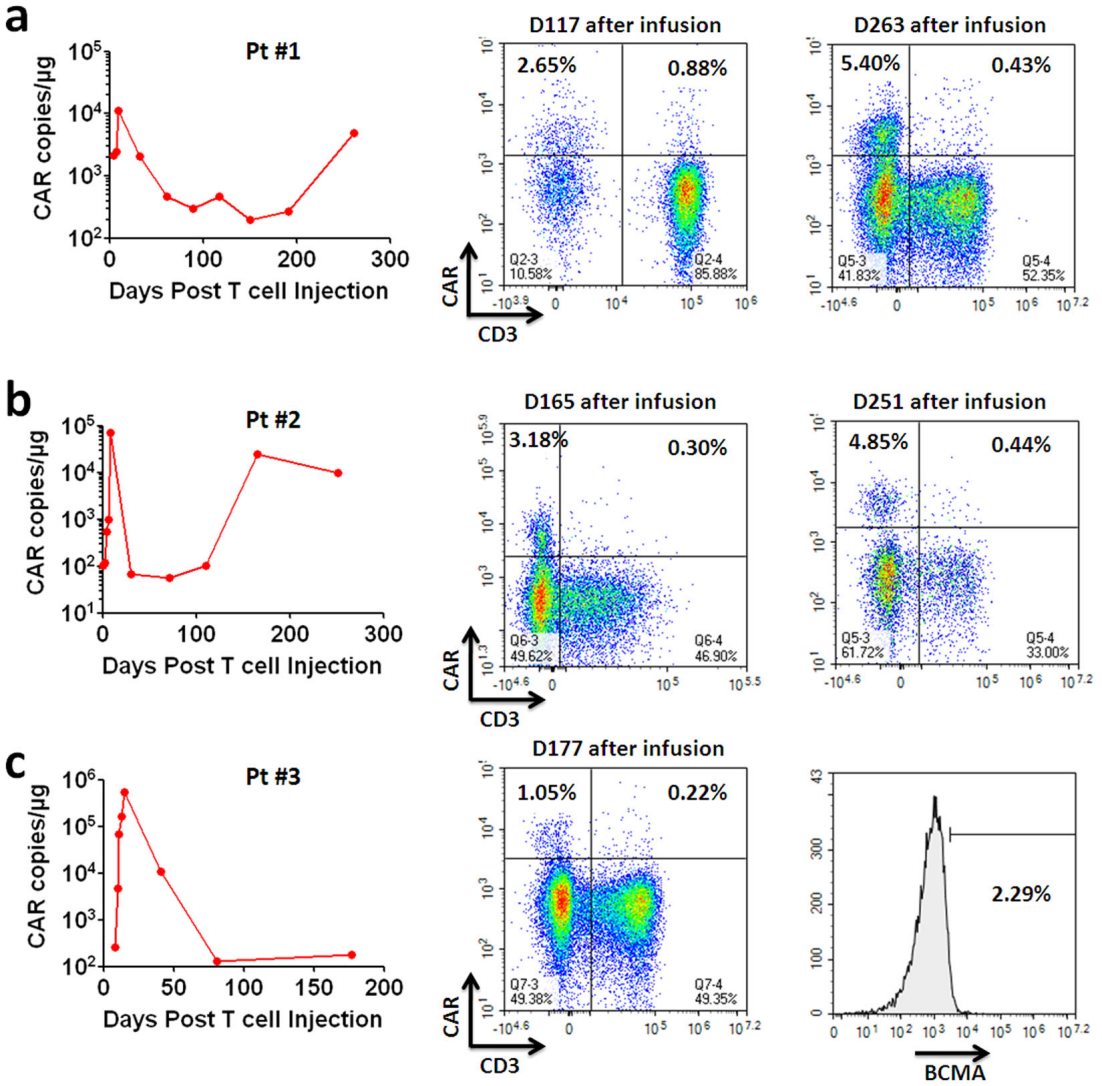
Persistence and re-expansion of CRISPR-edited anti-CD19 or anti-BCMA CART-aIL6/IL1RA with GM-CSF/TCR KO in patients. **a**–**c** Left panel, kinetics of CAR vector copies in the blood of treated patients and frequencies of CRISPR-edited CD3^–^ CART cells. Right panel, analysis of CD3^+^ and CD3^–^ CAR^+^ T cells in patient samples during long-term follow-up. **c** BCMA expression on lymphocytes of peripheral blood in patient #3 at D177 post infusion.

Analysis of CAR vector genomic copies in blood confirmed significant expansion of CART cells during therapy in all three patients (Fig. 5a–c). In patient #1, B-cell aplasia was maintained until follow-up examination at 3 months after infusion, however, CD19^+^ B-cell frequency bounced back to 7.83% (Fig. 4b). Interestingly, CART re-expanded slightly and eradicated B cells afterward. The analysis by flow cytometry revealed comparable re-expansion of CD3^–^ and CD3^+^ CART, suggesting that TCR KO did not impair the long-term persistence of CART cells. Similarly in patient #2, CRISPR-edited CD3^–^ CART cells re-expanded to high levels during antigen-positive relapse, confirming excellent persistence of CRISPR-edited CART cells. In patient #3, CRISPR-edited CD3^–^ CART cells did not re-expand probably because the relapsing tumor cells lost antigen expression, as suggested by the aberrant increase of urea κ LC with low levels of IgA (Fig. 4d) and no detection of BCMA^+^ cells in peripheral blood (Fig. 5c). These results suggest that CRISPR-edited GM-CSF/TCR KO CART cells were persistent after infusion and capable of re-expansion upon antigen exposure.

## Discussion

CART therapies have been approved for treating B-cell malignancy by FDA in the USA in 2017, and subsequently approved in clinics in Europe and Japan, with outstanding therapeutic efficacy. However, complete and durable remission in patients usually correlates with severe cytokine toxicity and neurotoxicity. Cytokine storm during CART therapy develops from two major sources, including the first wave of inflammatory cytokines secreted from CART and the second wave from patient immune cells. In this study, we demonstrated that CRISPR gene editing could effectively KO GM-CSF, which is not essential in CART therapeutic efficacy. Meanwhile, CART-secreted cytokine antagonists could automatically neutralize IL6 storm and block IL1 signaling by releasing high levels of anti-IL6 scFv and IL1RA. Our results indicated that the initial three treated patients experienced minimal cytokine toxicity and no neurotoxicity. With these encouraging preliminary results, we will continue enrolling more patients to validate the efficiency of CART-aIL6/IL1RA with GM-CSF/TCR KO for minimizing cytokine toxicity and neurotoxicity during CART therapy. As well, there are still many other cytokines significantly elevated during CART therapy, such as G-CSF, MCP-1, TNFA and MIP-1, IL8, IL13, and IL10. Further investigation is still needed to identify whether additional cytokines are critical in causing severe CRS and the source of such cytokines, which will allow optimization of the CART design for further development of truly safe CART cells with minimal cytokine toxicity.

Interestingly in patient 3, IL6 level dramatically increased after the peak of IFNG and IL1RA, and quickly decreased in the following 2 days. These observations implied that endogenous immune cells, such as macrophages and monocytes, were highly activated while CART was killing tumor targets. However, the IL6 produced by macrophages and monocytes was efficiently neutralized by CART-secreted anti-IL6 scFv during CRS. After eradication of tumor targets, macrophages and monocytes were still active in secreting IL6, but CART-secreted anti-IL6 blocker decreased significantly and could not efficiently neutralize the high level of IL6 after CRS ended. The transient IL6 increase after CRS did not cause any cytokine toxicity or neurotoxicity. On the other hand, the phenomenon in patient #3 suggested that IL6/IL1 blockers were secreted from CART cells in a transient manner only when CART cells were actively killing tumor targets, significantly relieving the concern of compromising patient immunity after CART treatment completed.

While our optimized CART design with CRISPR editing of cytokine and autonomous secretion of cytokine blockers significantly reduces cytokine toxicity and neurotoxicity, it is equally important that CART functions were not impaired and CRISPR editing would not cause safety concerns in treated patients. Our results demonstrated that GM-CSF/TCR KO CART did not show any property of tumor-prone transformation in mouse models or in the cancer patients. Recently, a study by Dr. Carl June’s team demonstrated that NY-ESO-1-specific T cells with CRISPR gene editing of TCR and PD-1 displayed robust antitumor activity and long-term safety in the treated patients, paving the path for wide application of CRISPR gene editing in T cell immunotherapy^2^. Their results also revealed that there was indeed low percentage of off-target cleavage events in the gene editing of TRAC, TRBC, and PD-1, and chromosome translocations. Despite of these unwanted genetic altercations in the T cells, there was no observation of the tumor-prone transformation of T cells in the treated patients. Consistently, our results further added clinical evidence to support the safety of CRISPR gene editing in CART therapy. In the current study, we have successfully and safely applied CRISPR gene editing to disrupt GM-CSF expression in CART cells co-expressing IL6 and IL1 blockers in treating patients with refractory hematologic malignancy. However, there are still several limitations with our current work, such as small patient numbers. It is also important to study the specific role of each cytokine self-blockade in reducing CRS. Furthermore, the identity of CRISPR-edited GM-CSF KO CART cells, such as their genetic landscape and memory T cell phenotype, warrants to be explored. In addition to safety concerns with CRS, it is still challenging to improve the long-term efficacy of CART therapy. Therefore, it would be of significance to investigate whether therapeutic efficacy of CRISPR-edited CART cells could be enhanced by novel strategies, such as transient delivery of modified telomerase reverse transcriptase (TERT) mRNA^8^.

Besides the risk of severe cytokine toxicity, another challenge of autologous CART therapy is the highly complicated and extremely expensive manufacturing process. Moreover, the quality of T cells from each cancer patient depends on a lot of parameters, which makes it difficult to guarantee the consistency of uniformly high-quality CART products for clinical treatment. In consideration of these challenges, there is an urgent need to develop off-shelf allogeneic CART cells for future. The fundamental risk about off-shelf allogeneic CART cells is graft-versus-host disease (GVHD), which can be significantly alleviated by KO of endogenous TCR in CART cells. The recent study by Dr. Carl June’s group successfully conducted CRISPR gene KO of endogenous TCR in T cells, in which subsequent substitution with antigen-specific TCR conferred the infused T with robust anti-tumor activity and long-term persistence^2^. Indeed, there has been evidence implying that TCR KO would not affect CART anti-tumor functions in mouse models^9,10^. Our study presents strong evidence that TCR KO did not impair CART proliferation, persistence, or anti-tumor functions in patients. In sum, our study provides a promising platform for reducing cytokine toxicity, from autologous or allogeneic settings.

## Materials and methods

### Clinical study design

The study was approved by the institutional ethics review committee of The Second Xiangya Hospital of Central South University. In brief, patients with refractory or relapsed Lymphoma or Multiple Myeloma were enrolled and peripheral blood was collected for CART ex vivo production. CAR was composed of the second generation 41BBζ signaling and extracellular antigen recognizing scFv derived from FMC63^11^ or C11D5.3^12^ targeting human CD19 or BCMA. During ex vivo expansion, transduced T cells were electroporated with sgRNA targeting GM-CSF (GCTCCCAGGGCTGCGTGCTG) and TRBC (GGAGAATGACGAGTGGACCC) and Cas9 RNP by BTX ECM830. The prior treatment patients had and the disease status at the time of CART cell infusion are included in Table 1. Before infusion, Patients #1 and #2 did not receive chemotherapy to lower tumor burden. Patient #3 showed circulating BCMA^+^ tumor cells in peripheral blood and, therefore, received treatment of Bortezomib, Pegylated liposomal Doxorubicin, and Dexamethasone (PDD regimen). By hematoxylin-eosin (HE) staining, the percentage of plasma cells was 80% and 84% in the bone marrow before and after PDD treatment. IgA level was 60.3 g/L and 40.5 g/L before and after PDD treatment, suggesting a partial reduction of tumor burden after the PDD regimen and before CART infusion. Before infusion, all patients were pretreated with lymphodepletion regimen F/C. After infusion with CART-aIL6/IL1RA with GM-CSF/TCR KO, patients were monitored for clinical signs of CRS and examined for clinical response of tumor remission. CRS was graded according to the ASTCT CRS Consensus Grading criteria as referenced^13^.

### sgRNA screening

sgRNA targeting the first exon of gene encoding GM-CSF was designed and synthesized according to the protocol described in the kit (Thermo Fisher). Primary T cells from a healthy donor (purchased from vendor PPA research) were activated with anti-CD3&CD28 dynabeads (Thermo Fisher) and then electroporated with RNP complex of the sgRNA candidates and Trucut V2 Cas9 protein (Thermo Fisher). Afterward, T cells were analyzed by intracellular staining of cytokines (LSR II, BD) to assess the efficiency of gene editing. The sgRNA targeted sequences before PAM motif are listed as:

1 GCTGCAGAGCCTGCTGCTCT; 2 GGAGCATGTGAATGCCATCC;

3 GCATGTGAATGCCATCCAGG; 4 GAGACGCCGGGCCTCCTGGA;

5 GATGGCATTCACATGCTCCC; 6 GCTCCCAGGGCTGCGTGCTG;

7 GCGTGCTGGGGCTGGGCGAG; 8 GCTGGGGCTGGGCGAGCGGG.

### T-cell gene editing and ex vivo expansion

Peripheral blood was collected from the enrolled patient and processed with Ficoll (GE Healthcare) gradient centrifugation to isolate PBMC. T cells were purified from PBMC with the pan T isolation kit (Miltenyi). Purified T cells were activated with anti-CD3&CD28 dynabeads (Thermo Fisher) and transduced with 3rd generation lentivector encoding anti-CD19 or anti-BCMA CAR co-expressing IL6/IL1 blockers (scFv derived from Sirukumab and IL1RA), followed by electroporation with RNP complex of the sgRNA (targeting GM-CSF and TRBC) and Trucut V2 Cas9 protein (Thermo Fisher). T cells were further ex vivo expanded and analyzed for CAR expression and gene editing efficiency (Novocyte, Agilent). The CART cells were also tested for sterility and in vitro killing of leukemia cells Nalm6 (ATCC) expressing GFP or RPMI-8226 (ATCC) cells (Novocyte, Agilent).

### Flow cytometry analysis of CAR expression

CAR was stained by a primary biotinylated goat-anti-mouse Fab antibody and secondary PE-Strepavidin (Jackson Immune), followed by analysis on Novocyte.

### Western blotting analysis

Cell culture supernatant was mixed with 6× SDS reducing buffer and boiled for 5 min. The reduced samples were then loaded into the wells of 10% sodium dodecyl sulfate-polyacrylamide gels for electrophoresis which was carried out at 80 V for 0.5 h and then at 120 V for 1 h, and the protein gel was transferred to PVDF membrane (Millipore, Bedford, MA). The membrane was blocked in 5% skim milk for 1.5 h at room temperature, followed by incubation for overnight at 4 °C with specific primary antibodies (anti-human IL1RA: 1:2000 dilution, Abcam; and His-tag: 1:1000 dilution, Cell signaling). After washing with TBST buffer three times (10 min each), the membrane was incubated with appropriate secondary antibodies conjugated to horseradish peroxidase (1:10,000 dilution, Proteintech) at room temperature for 1 h. Proteins were then detected using enhanced chemiluminescence reagents (NcmECL Ultra).

### Analysis of cytokines by Elisa

The peripheral blood samples were collected from infused patients. The plasma samples were analyzed by Elisa kits (Boster) according to manufacturer’s protocol to measure the concentrations of cytokines during treatment.

### Quantitative PCR

Genomic DNA was purified from a patient blood sample by gDNA isolation kit (Thermo Fisher), and analyzed with SYBR Green qPCR kit (Takara) according to manufacturer’s protocol on Lightcycler (Roche).

### Animal study

Celledit had a working agreement with WPI and animal study was approved by the WPI IACUC committee. 6–8 week NSG mice (JAX) were intravenously injected with 1 × 10^6^ Nalm6 leukemia cells modified to stably express GFP. Six days later, the mice were intravenously injected with 2 × 10^6^ GM-CSF/TCR KO or WT anti-CD19 CART, which also express IL6/IL1 blockers (*n* = 6 for GM-CSF KO, or *n* = 5 for GM-CSF WT). Mice not receiving CART cells were included as a control (indicated “CTRL” in the figures, *n* = 4). Post T-cells injection, the mice were monitored for body weight, survival, and the number of GFP^+^ Nalm6 leukemia cells and CD45^+^CD3^–^ T cells in the blood by Trucount beads (BD Biosciences).
